# Improving dental implant design through a combined analysis of the insertion process and push-in test

**DOI:** 10.1038/s41598-026-53352-3

**Published:** 2026-05-18

**Authors:** Armin Shanazari, Gholamreza Rouhi

**Affiliations:** Faculty of Biomedical Engineering, Amirkabir University of Technology, Tehran, Iran

**Keywords:** Dental implant, Primary stability, Finite element method, Insertion torque, Insertion energy, Bone-implant strength, Engineering, Health care, Medical research

## Abstract

This study examined the influence of key dental implant design parameters, including taper angle, thread thickness, thread pitch, and number of thread starts, on the primary stability (PS) indicators derived from insertion process and push-in test. Finite element models of nine implant designs, including a baseline and eight single-parameter variations, were developed. Simulations of implant insertion were performed to evaluate maximum insertion torque (MIT) and insertion energy (IE), followed by push-in tests to assess bone–implant construct stiffness and strength. Linear regression analysis quantified the influence of each design parameter on the PS indicators and examined interrelationships among PS indicators. The results showed that PS indicators are affected in a complex, parameter-specific manner. Increasing taper angle increased MIT while decreasing IE, with minimal effects on stiffness and strength, whereas increasing thread thickness increased both MIT and IE but reduced construct stiffness and strength. No overall linear relationships were observed among PS indicators; however, variations in thread thickness and pitch produced strong linear relationships between MIT and IE (R² = 0.93 and 0.94, respectively). By jointly considering the insertion process and push-in test through linear regression analysis, an improved implant design was identified, resulting in 17% higher MIT and 10% greater bone–implant construct strength.

**Supplementary Information:**

The online version contains supplementary material available at 10.1038/s41598-026-53352-3.

## Introduction

Dental implants are commonly used to replace missing teeth, providing a long-term solution to restore oral function. The performance of dental implants is primarily evaluated based on the concept of stability, which is categorized into primary and secondary stability^1^. Primary Stability (PS) refers to the initial mechanical engagement of the implant with the surrounding bone immediately after insertion. This engagement is purely mechanical, influenced by different factors, such as bone quality, implant design, and the surgical technique, with no involvement of biological factors^2,3^. Secondary stability, on the other hand, is developed over time as the bone heals and forms new bony tissue around the implant, which is heavily influenced by biological factors^1,4^.

Achieving optimal secondary stability necessitates adequate maximum insertion torque (MIT), which helps prevent excessive micromovement between the implant and bone, and thus ensures proper integration and avoids complications^3,5^. In clinical practice, MIT is often assessed through non-invasive tests, such as insertion torque measurement and resonance frequency analysis (RFA)^6^. The MIT refers to the maximum value of torque applied during the implant insertion, which is a commonly employed indicator of PS^7^. Additionally, insertion energy (IE), which is derived from the area under the insertion torque versus rotation angle curve, has been proposed as another important measure of PS in various studies^8,9^ to date.

In in-vitro testing conditions, implant stability can be evaluated by assessing the mechanical properties of the bone-implant construct, specifically its stiffness and holding power, which correspond to reversible and irreversible deformations, respectively^10,11^. Mechanical tests, such as push-in and pull-out tests, apply axial compressive or tensile forces, respectively, to the bone-implant structure until failure occurs. The maximum force that the bone-implant system can withstand, as well as the slope of the force-displacement curve in the elastic region, are used to define the stiffness and strength (holding power) of the bone-implant construct^12,13^.

The insertion process plays a crucial role in the implantation process, especially in immediate loading treatments, where a high MIT is required^14,15^. Immediate loading treatment method reduces the time required for the treatment process, and intends to enhance the patient’s quality of life. However, in individuals with a low bone quality, the use of immediate loading is not feasible, which can be deemed as a significant challenge. While the MIT is generally dependent on bone volume fraction and quality, other factors, such as surgical technique and implant macro-geometry can play their crucial roles^16^. By modifying the two aforementioned factors, i.e., surgical technique and implant macro-geometry, the MIT can be manipulated.

One of the methods to increase the MIT is to reduce the pilot-hole diameter, a process known as osteotomy underpreparation^17–20^. Ovesy et al.^20^ compared the insertion process and bone-implant structure stiffness in two different osteotomy methods: one with a smaller pilot-hole for a soft bone, and another with a larger pilot-hole for a dense bone. Their results showed that smaller pilot-holes increased insertion torque, but did not significantly affect the bone-implant stiffness. Additionally, Degidi et al.^19^ demonstrated that reducing the pilot-hole diameter increases MIT and IE, but has no effect on the RFA. In this regard, other studies have shown that reducing the pilot-hole diameter can only increase MIT in the apical region, with no impact on RFA^17,18^. However, Cha et al.^21^ have shown that the increase in MIT resulting from underpreparation could cause damage production in the peri-implant bone tissue and thus can lead to bone loss, as confirmed by animal studies^22,23^.

Based on the concern raised regarding potential damage caused by underpreparation, another solution for increasing the MIT can be making a modification in implant macro-geometry design. To date, various studies have investigated the effects of implant macro-geometry design on the insertion process. For instance, Wu et al.^24^ compared the insertion process of cylindrical and conical implants and showed that conical implants provide a higher MIT compared to cylindrical implants. In another effort, Wang et al.^9^ compared the insertion processes of three dental implants with completely different geometries under in-vitro conditions, focusing on MIT and IE. They observed that implant design significantly influenced the MIT and IE, but had little impact on the RFA^9^. Moreover, Yamaguchi et al.^25^ examined the effects of thread pitch and a double-threaded design on dental implants under in-vitro conditions, and showed that reducing the thread pitch/lead increases the MIT. In another study, Grobecker-Karl et al.^26^ studied the effects of implant length and diameter on the insertion process by comparing an implant with a shorter length and a smaller diameter to a standard implant. They found that reducing the length and diameter of the implant significantly decreased IE, but had no notable effect on the MIT. Taken together, these studies indicate that modifications in implant macro-geometry, such as implant body shape^24^, thread pitch/lead^25^, and implant dimensions^26^ can substantially influence the implant insertion process and its related indicators, particularly MIT and IE^9^.

One of the gaps in the current literature is the limited investigation of the parametric effects of different implant designs on the insertion process, likely due to the high cost of both manufacturing implants with varied geometries and the in-vitro tests requirements. Nonetheless, the use of finite element modeling (FEM) has allowed researchers to simulate the insertion process, as well as to mimic mechanical tests, such as push-in test, which provides a cost-effective alternative, while one can control the effects of various parameters involved in the experiments. So far, in-silico studies using homogenized and micro FEM have successfully modeled the dental implant insertion process, while considering the plasticity and damage properties of the bone tissue^20,27–29^. However, these studies have been limited to examining the effects of bone density and surgical techniques on the insertion process, and to date, no in-silico research has explored the impact of dental implant geometry on the insertion process, to the best of our knowledge. Furthermore, implant geometry represents one of the key parameters that can be engineered and modified to improve clinical performance of dental implants. Therefore, a comprehensive investigation is required to clarify how different type of variations in implant design influence the insertion process, and how these changes may subsequently affect the mechanical performance of the bone–implant construct.

Based on the literature review made in this study and the identification of existing research gaps, this work aimed to investigate the effects of dental implant design parameters on the insertion process, focusing on PS indicators, such as MIT and IE, as well as on the mechanical properties of the bone-implant construct in push-in tests, including its stiffness and strength. Using a finite element model, the insertion process and push-in test were simulated consecutively for implants with varied designs, allowing for a systematic evaluation of the main design parameters’ effects on PS indicators. Based on these analyses, finding a modified implant design was another goal of this work, which was validated through various simulations, with an improved performance in both insertion process and push-in test.

## Materials and methods

### Modeling implant geometry and experimental design

#### Base model design

**Fig. 1:**
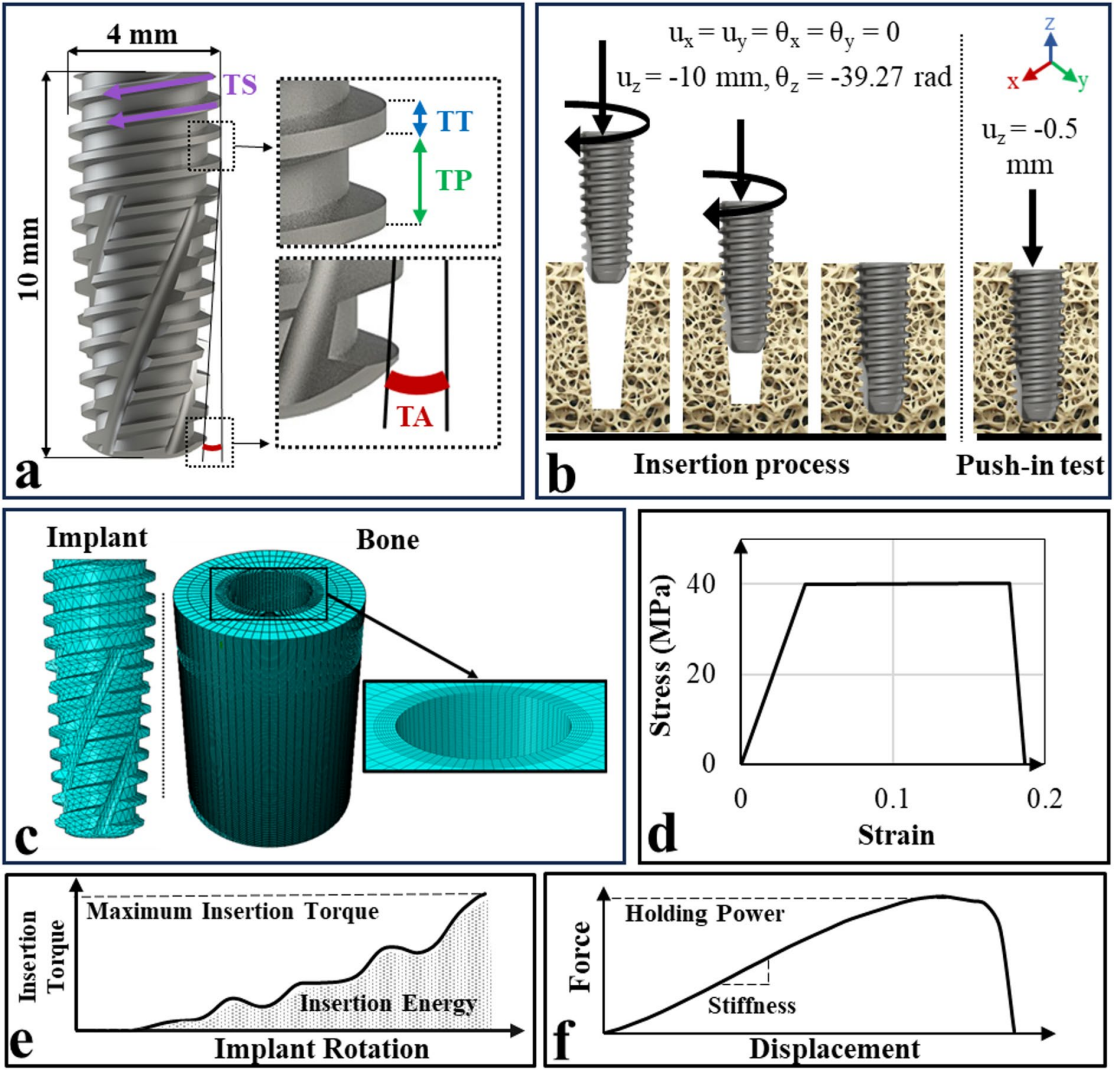
Schematic representation of the methodology used in this study: (**a**) Geometry design of the base model, (**b**) Boundary conditions employed in the insertion process and push-in test, (**c**) Mesh of the base model implant and bone geometry, (**d**) Stress-strain curve related to bone material properties, (**e**) Insertion torque versus implant rotation resulted from the insertion process, (**f**) Force versus displacement curve resulted from the push-in test.

To investigate the effects of implant design on its performance, a base model of a dental implant was developed, which serves as a reference for assessing the impact of design parameters on implant performance. The base implant model in this study was designed based on an implant from Cowellmedi company (South Korea). To standardize the implant geometry, the thread profile was designed to be trapezoidal, with design parameters selected to be at the midpoint between the minimum and maximum values based on prior literature^30,31^(Fig. 1a; Table 1).

#### Experimental design and number of simulations

The effects of design parameters on implant performance were examined using the one factor at a time (OFAT) method. This method, selected for its simplicity and focus on the direct effects of each parameter, involved varying one parameter at a time across three levels: minimum (Lowest), base (Base), and maximum (Highest), while keeping all other parameters constant. The design parameters investigated here include: taper angle (TA), thread thickness (TT), thread pitch (TP), and the number of thread starts (TS). Table 1 presents the minimum and maximum values for the design parameters of interest, along with the midpoint values that were selected for the base model in the present study.

**Table 1:** The maximum and minimum values for the parameters studied in this research.

	Parameter Levels		
	Lowest	Highest	Mid (base)
Taper Angle (TA, degree)	0	6	3
Thread Thickness (TT, mm)	0.1	0.3	0.2
Thread Pitch (TP, mm)	0.55	1.05	0.8
# of Threads starts (TS)	Single-Threaded	Triple-Threaded	Double-Threaded

Given that four design parameters were evaluated and three levels were assigned to each, a total of 9 experiments were conducted, ensuring the ability to examine the individual and precise effects of each parameter. For each parameter, two experiments were conducted: one with the minimum value and the other with the maximum value. Along with the base model, this design structure provided three different implant designs per parameter, with other parameters held constant. The design parameters for each of the nine experimental setups are shown in Table 2.

**Table 2:** Design parameters for all experiments conducted in this study.

	Design Parameters			
	Taper Angle (TA, degree)	Thread Thickness (TT, mm)	Thread Pitch (TP, mm)	# of Thread Starts (TS)
Base Model	3	0.2	0.8	2
Lower TA	6	0.2	0.8	2
Higher TA	0	0.2	0.8	2
Lower TT	3	0.1	0.8	2
Higher TT	3	0.3	0.8	2
Lower TP	3	0.2	0.55	2
Higher TP	3	0.2	1.05	2
Single-Threaded	3	0.2	0.8	1
Triple-Threaded	3	0.2	0.8	3

### Finite element modeling of the insertion process and push-in test

Finite element simulations were conducted using Abaqus 6.14 (Dassault Systems, France). The explicit dynamic solver was selected due to the highly nonlinear and dynamic nature of the implant insertion- and push in processes, which involve complex contact interactions, large deformations, and progressive material damage. This solver has been widely used in some other studies, which investigated similar implant–bone interactions due to its numerical stability in simulations, involving severe element distortion and element deletion^20,27,29,32^.

For each implant design, a finite element simulation comprising an insertion process and a push in test was performed. Each simulation included one implant and one bone block, with the bone represented as a cylinder with a diameter of 7.2 mm and a height of 10 mm. At the start of the analysis, the implant was positioned directly above the pilot hole without initial contact. The pilot-hole geometry matched the internal shape of each implant (i.e., the implant geometry excluding threads) to avoid intentional pilot-hole undersizing or oversizing. Accordingly, except for the 0° and 6° taper angle designs, the pilot hole was modeled as a cylindrical–conical cavity with a 3.2 mm cylindrical diameter and a 3° conical angle. For the implant with a 0° taper angle, the pilot hole was modeled as a simple cylinder with a 3.2 mm diameter, whereas for the 6° taper angle implant, a cylindrical–conical hole with a 3.2 mm cylindrical diameter and a 6° taper angle was considered.

#### Material properties modeling

Due to the significant difference in elastic properties between titanium and trabecular bone, the implant was modeled as a rigid body in all simulations, similar to some previous research^20,27,32^, and its density was defined as 4.43 g/cm³^32^. The bone, exhibiting nonlinear and damage-prone behavior, was modeled using an elastoplastic material model with damage, which is common in the current literature^20,27,32^. The von Mises yield criterion with a yield stress of 40 MPa was used to simulate bone plasticity, corresponding to bone with a volumetric fraction of 40–45%^33^. Additionally, a ductile damage model was employed, where elements were removed after 10 μm of displacement^27,32,34^. The mechanical properties of spongy bone are provided in Table 3, and the stress-strain curve for the bone material is shown in Fig. 1d.

**Table 3:** Material properties of trabecular bone used in this research (Dorogoy et al.^32^ and Guan et al.^35^).

	Material Property Constants					
	Density (gr/cm^3^)	Young’s Modulus (MPa)	Poison’s ratio	Yield Stress (MPa)	Plastic Fracture Strain	Damage Evolution (µm)
Trabecular Bone	1	700	0.35	40	0.135	10

#### Boundary conditions and loading

Each implant sample was simulated in two stages: the insertion process and the push-in test. A rest period was defined between the two stages to eliminate the dynamic effects from the explicit solver during the insertion process. The loading was applied through a reference point kinematically connected to the implant body. The lateral surfaces of the bone model were fully constrained in all degrees of freedom. A friction coefficient of 0.61 was assumed for the contact properties between the implant and bone^27,32,35^. Due to the potential removal of bone elements during the insertion and push-in tests, a general contact model was used in the explicit solver of Abaqus software^34^.

For the ideal insertion simulation, the rotational movement of each implant was considered based on its thread lead, ensuring that with each full rotation, the implant undergoes a translational axial displacement equal to one thread lead into the bone. For example, in the base model, a translational displacement of 10 mm (implant length) and a rotation of 39.27 radians (based on a 1.6 mm thread lead) were applied to the implant’s reference point (Fig. 1b). The thread lead and corresponding rotation required to reach the final insertion depth for all implant designs investigated in this study are reported in Supplementary Table S.2.

In the push-in test simulation, axial displacement loading was applied at a constant speed of 0.5 mm per second, with a total displacement of 0.5 mm exerted on the reference point^20^, ensuring that failure occurred within the prescribed displacement range for all models and that the peak force (strength) could be captured. To ensure quasi-static conditions, the ratio of kinetic energy to internal energy was monitored throughout the simulation and remained below 5%^36^.

#### Mesh generation and mesh sensitivity analysis

To minimize computational cost, while maintaining the accuracy of the model, the bone geometry was meshed such that finer elements were placed near the implant and larger elements were used farther away (Fig. 1c). To assess model sensitivity to mesh size, the bone geometry was discretized using 8-node hexahedral elements with element sizes of 200, 100, 50, and 25 μm. The optimal mesh size of 50 μm was selected after conducting mesh sensitivity analysis (see Supplementary Note: Mesh Independence Analysis, and Supplementary Figs. S.1 and S.2). Since the implant was modeled as a rigid body and therefore did not undergo deformation, the discretization primarily served to represent its external geometry. Accordingly, the implant geometries were discretized using 10-node tetrahedral elements, allowing accurate representation of the complex threaded geometry and sufficient nodal resolution on the implant surface. The bone model consisted of 228,800 elements and 250,848 nodes, which remained identical for all models in this study. The base model implant consisted of 23,037 elements and 35,229 nodes. Mesh characteristics corresponding to the remaining geometries investigated in this study are provided in Supplementary Table S.2.

### Data extraction and analysis

#### Data extraction

From the insertion process simulations, the applied torque curve versus implant rotation was extracted, and the MIT and IE values were calculated and considered as PS indicators (Fig. 1e). In the push in test, the force–displacement curve was extracted as the output. The strength of the implant–bone construct was defined as the peak force of this curve, while stiffness was calculated as the slope of the curve in the elastic region (Fig. 1f).

#### Impact coefficient analysis

To quantify the influence of each design parameter on PS indicators, a linear regression analysis was performed. All data were first normalized to a scale of 0 to 1, using min-max normalization to ensure comparable scales across different input and output parameters. For each design parameter, three data points were available corresponding to the minimum, medium, and maximum levels. Linear regression was applied to these three points to establish the relationship between each design parameter and each PS indicator. The slope of the regression line was calculated as the impact coefficient, representing the magnitude and direction of influence of each parameter on the PS indicators.

#### Relationship between stability indicators

Given that the MIT, IE, and implant-bone construct strength and stiffness were considered as indicators of the PS, it is essential to examine the relationships among these indicators. To examine the linear relationship, scatter plots were generated for each pair of PS indicators using the complete dataset. Each indicator was standardized by dividing its value by its respective mean, thereby preserving the data distribution and relative scatter. Linear regression lines were fitted to each scatter plot, and the coefficient of determination (R²) was calculated to quantitatively assess the degree of linearity. This analysis was selectively performed for specific groups of experiments where the investigation of inter-indicator linearity was deemed necessary, as well as for the complete dataset.

#### Improved design

Based on the impact coefficients determined in the previous analysis, an improved implant design was developed. For each design parameter, the level that produced the most favorable impact on PS indicators was selected. When a design parameter exhibited both positive and negative effects across the stability indicators, its value was set to the level (maximum or minimum) at which the sum of positive impact coefficients outweighed the sum of negative ones. Conversely, if the negative impacts were dominant, the parameter was set to its lowest level. This decision was based on the calculated impact coefficients of the parameter for each stability indicator.

#### Model validation and performance evaluation

The improved implant design was validated through simulations of both the insertion process and push-in test protocols, which were used for the baseline model. The torque-penetration relationship was analyzed and compared with the baseline design to evaluate improvements in MIT and IE. Force-displacement curves were generated and analyzed to assess changes in implant-bone construct stiffness and strength. Quantitative metrics, including percentage changes in MIT, IE, construct stiffness, and construct strength were calculated. Direct numerical comparison was performed between the improved design and baseline model outputs to quantify the enhancement achieved through the optimization process.

## Results

The results of the primary stability indicators, i.e., maximum insertion torque (MIT), and insertion energy (IE) obtained from the insertion process simulations, as well as the stiffness and strength of the bone-implant construct derived from the push‑in test simulations, for each dental implant design investigated here can be found in Table 4. The relative percentage changes in all outcome measures with respect to the base model are also reported in this table.

**Table 4:** Summary of all the primary stability indicators (maximum insertion torque (MIT), insertion energy (IE), bone-implant stiffness, and strength) for the baseline model and eight experimental design models, with their percentage differences relative to the baseline model.

	Simulation Outputs on Primary Stability Indicators					
	Insertion Process			Push-in Test		
	MIT (*N*·cm)		IE (J)	Strength (kN)		Stiffness (kN/mm)
Base Model	29.14		3.89	0.99		5.56
Lower TA	16.37 (-44%)		4.32 (11%)	1.01 (2%)		5.65 (2%)
Higher TA	36.20 (24%)		2.89 (-26%)	0.97 (-2%)		5.48 (-1%)
Lower TT	26.64 (-9%)		3.03 (-22%)	1.12 (13%)		5.85 (5%)
Higher TT	33.57 (15%)		4.21 (8%)	0.82 (-17%)		5.22 (-6%)
Lower TP	33.39 (15%)		6.21 (59%)	0.81 (-18)		5.45 (-2%)
Higher TP	23.59 (-19%)		2.62 (-44%)	0.95 (-4%)		5.31 (-4%)
Single-Threaded	25.34 (-13%)		5.92 (52%)	0.98 (-1%)		5.54 (0%)
Triple-Threaded	33.15 (14%)		3.14 (-19%)	1.03 (4%)		5.61 (1%)

### Results corresponding to the insertion process

**Fig. 2:**
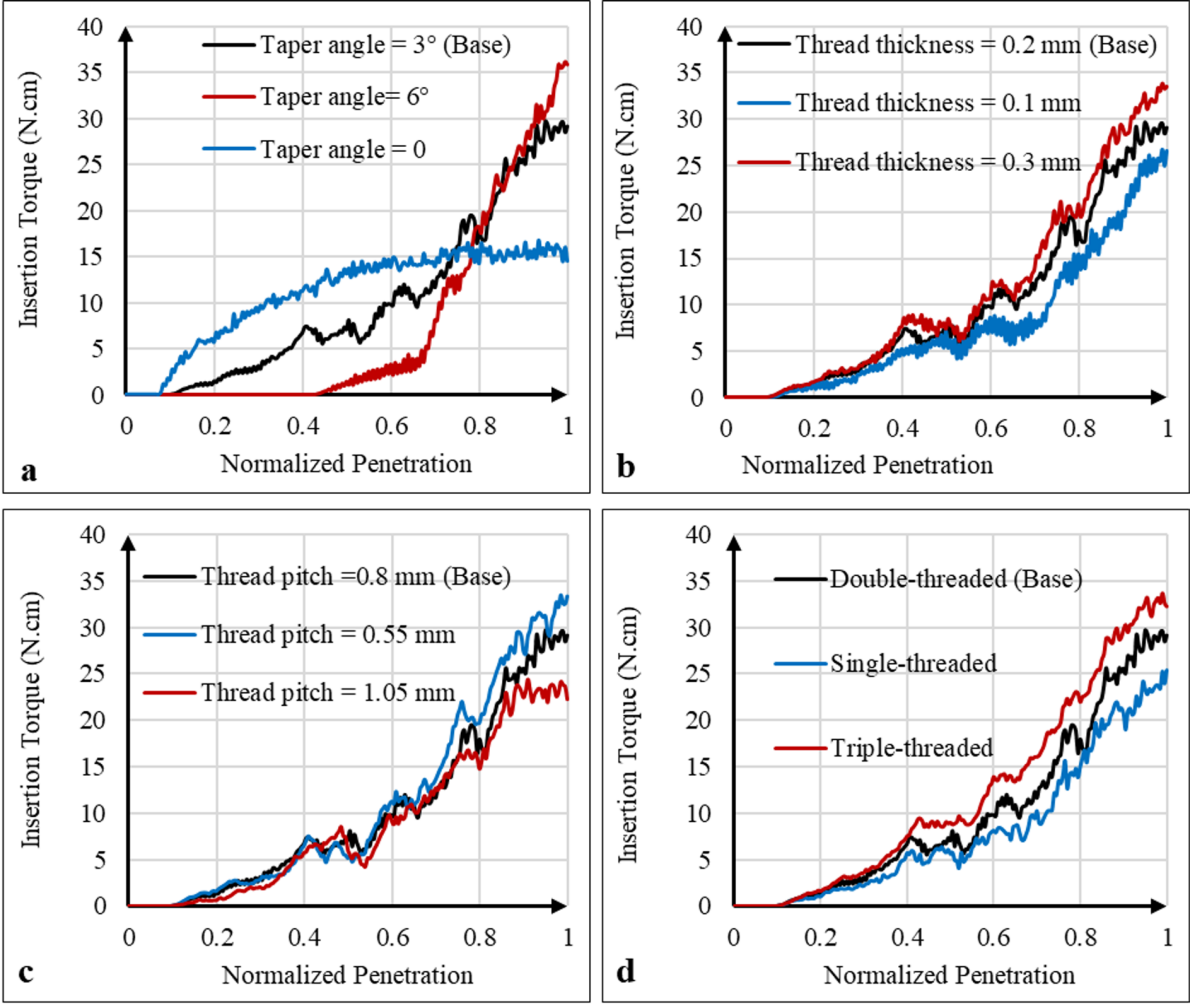
Normalized insertion torque versus penetration depth curves resulting from finite element simulations of the insertion process: (**a**) Taper angle parameter group, (**b**) Thread thickness parameter group, (**c**) Thread pitch parameter group, and (**d**) Number of thread starts parameter group.

As can be seen in Fig. 2, variations in implant design parameters produced distinct effects on the insertion torque trend, as well as on it maximum value (MIT). For instance, increasing the taper angle from 3° (base model) to 6° resulted in a 24% increase in MIT, whereas reducing the taper angle from 3° to 0° led to a 44% decrease in MIT. Similarly, increasing thread thickness from 0.2 mm to 0.3 mm increased MIT by 15%, while a reduction from 0.2 mm to 0.1 mm decreased MIT by 9%. In contrast, increasing the thread pitch from 0.8 mm to 1.05 mm reduced MIT by 19%, whereas decreasing the pitch from 0.8 to 0.55 mm increased MIT by 14%. With respect to threading configuration, the triple‑threaded design increased MIT by 13% relative to the double‑threaded base model, while the single‑threaded design resulted in a 15% reduction. On the other hand, the insertion energy (IE) exhibited different trends from those observed for MIT. Increasing the taper angle and the number of thread starts resulted in a reduction in IE, whereas increasing thread thickness and thread pitch led to higher IE values. The magnitudes of both MIT and IE changes for each design variation, relative to the base model, can be seen in Table 4.

### Results corresponding to the bone-implant construct push-in process

**Fig. 3:**
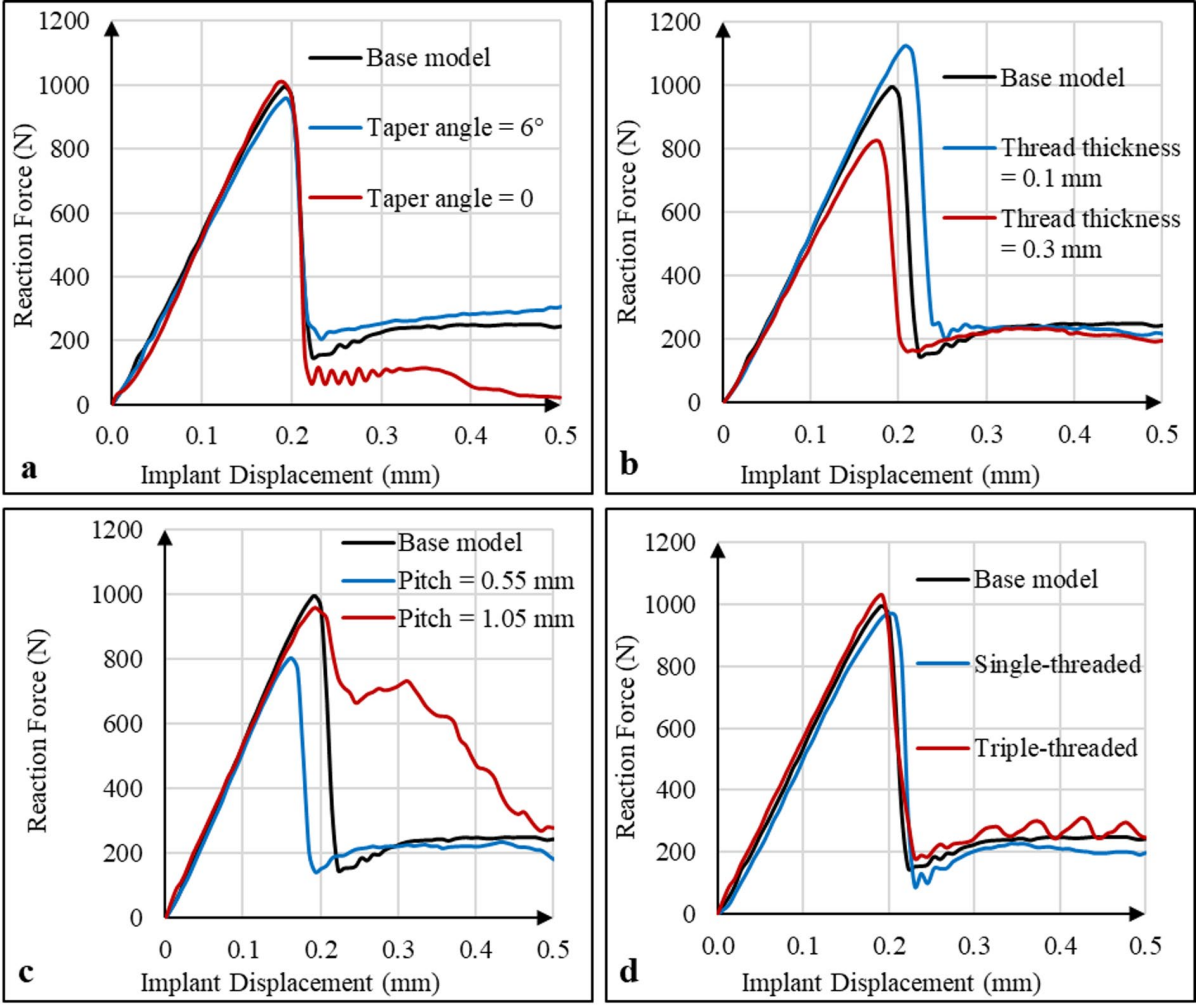
Force-displacement curves resulted from finite element simulation of bone-implant construct push-in tests: (**a**) Taper angle parameter group, (**b**) Thread thickness parameter group, (**c**) Thread pitch parameter group, and (**d**) Number of thread starts parameter group.

The push‑in test results indicated that variations in taper angle and number of thread starts had a negligible effect on the force–displacement response of the bone-implant construct (Fig. 3a and d). In contrast, thread thickness markedly influenced the bone-implant construct strength (Fig. 3b), such that increasing the thread thickness from 0.2 mm (base model) to 0.3 mm reduced the construct strength by 17%, whereas decreasing from 0.2 mm to 0.1 mm resulted in a 13% increase in the strength. Furthermore, both reduction and increase in the thread pitch, relative to the base design, led to a reduction in implant–bone construct strength (Fig. 3c). To be more specific, an increase and a reduction in the thread pitch by 0.35 mm decreased the strength by 4% and 18%, respectively. Regarding structural stiffness of the bone-implant construct, all investigated design parameters exhibited trends consistent with those observed for strength.

### The effects of design parameters on primary stability indicators

**Fig. 4:**
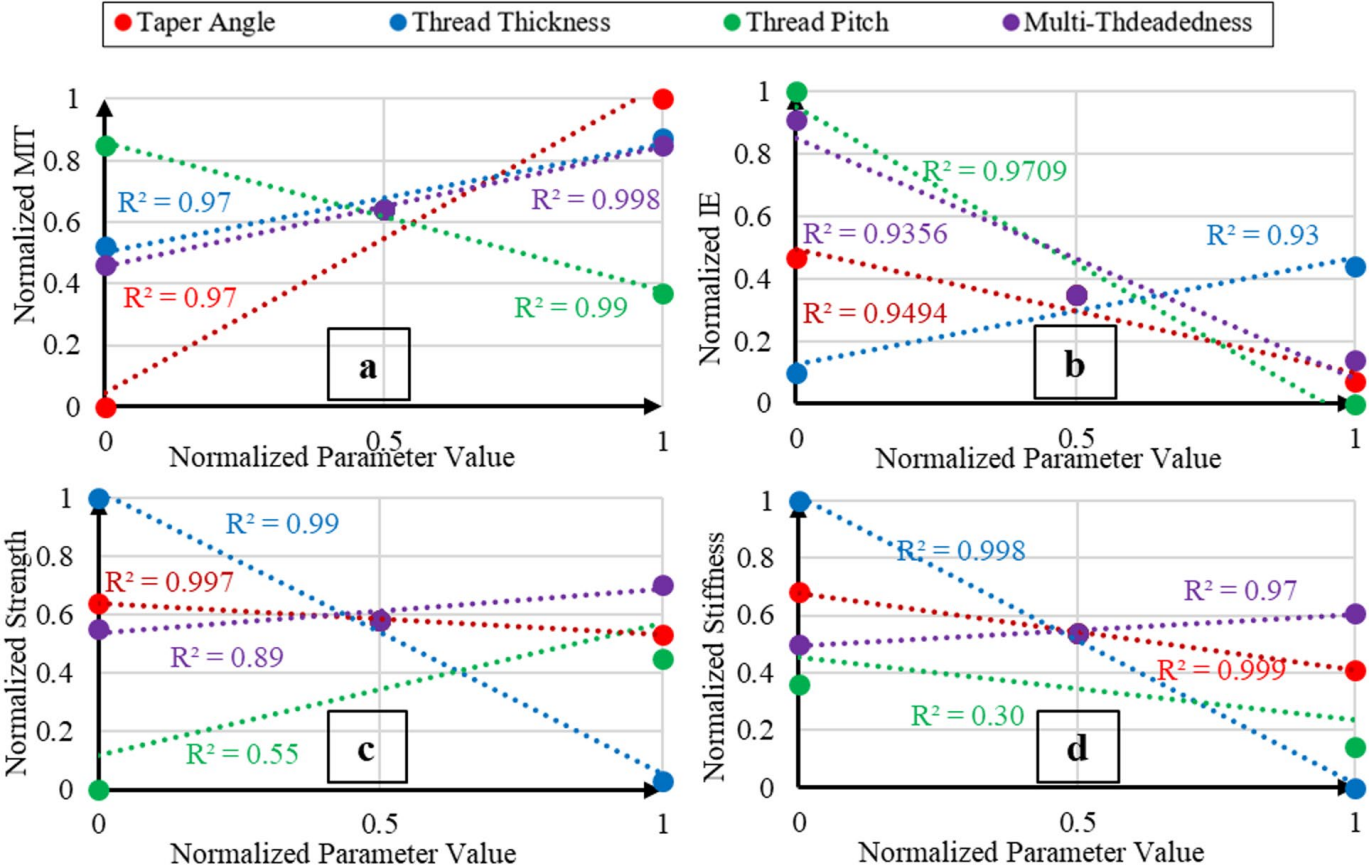
Effects of design parameters on the primary stability indicators of dental implant: (**a**) Normalized MIT, (**b**) Normalized IE, (**c**) Normalized strength of bone-implant, and (**d**) Normalized stiffness of bone-implant.

The effects of normalized design parameters variations on the primary stability indicators are presented in Fig. 4, and the corresponding impact coefficients are summarized in Table 5. Since there was no meaningful linear relationship between thread pitch and strength, as well as stiffness (R² = 0.55 and 0.30, respectively, in Fig. 4c and d), the associated impact coefficients were not reported in Table 5. The taper angle exhibited the strongest positive impact, whereas thread pitch imposed the strongest negative effect on MIT (Fig. 4a; Table 5). Moreover, IE was most strongly increased by thread thickness, while thread pitch showed the greatest negative influence on it. (Fig. 4b; Table 5). For the push-in test responses, thread thickness had the most pronounced negative impact on both bone-implant construct stiffness and strength, while the number of thread starts showed a negligible effect on these two indicators (Fig. 4b and c, and Table 5).

**Table 5:** Impact coefficients of each design parameter on the primary stability indicators, calculated using linear regression analysis for all simulation conditions.

	Coefficient of Impacts on Primary Stability Indicators			
	Insertion process		Push-in test	
	MIT	IE	Strength	Stiffness
Taper angle	1.00	-0.40	-0.11	-0.27
Thread thickness	0.35	0.34	-0.100	-1.00
Pitch	-0.48	-1.00	-	-
Multi-threadedness	0.39	-0.77	0.15	0.11

### Relationship between different primary stability indicators

**Fig. 5:**
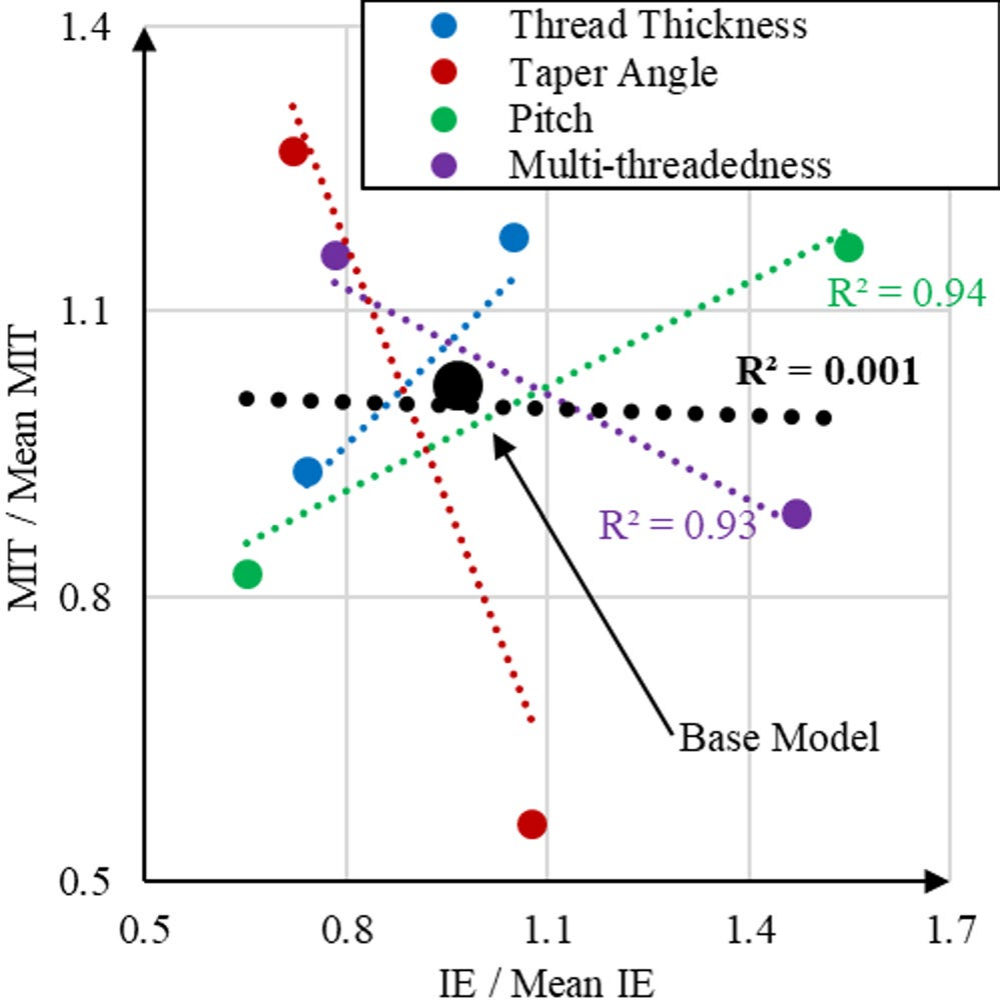
Normalized MIT versus normalized IE parameter for different values of design parameters.

**Fig. 6:**
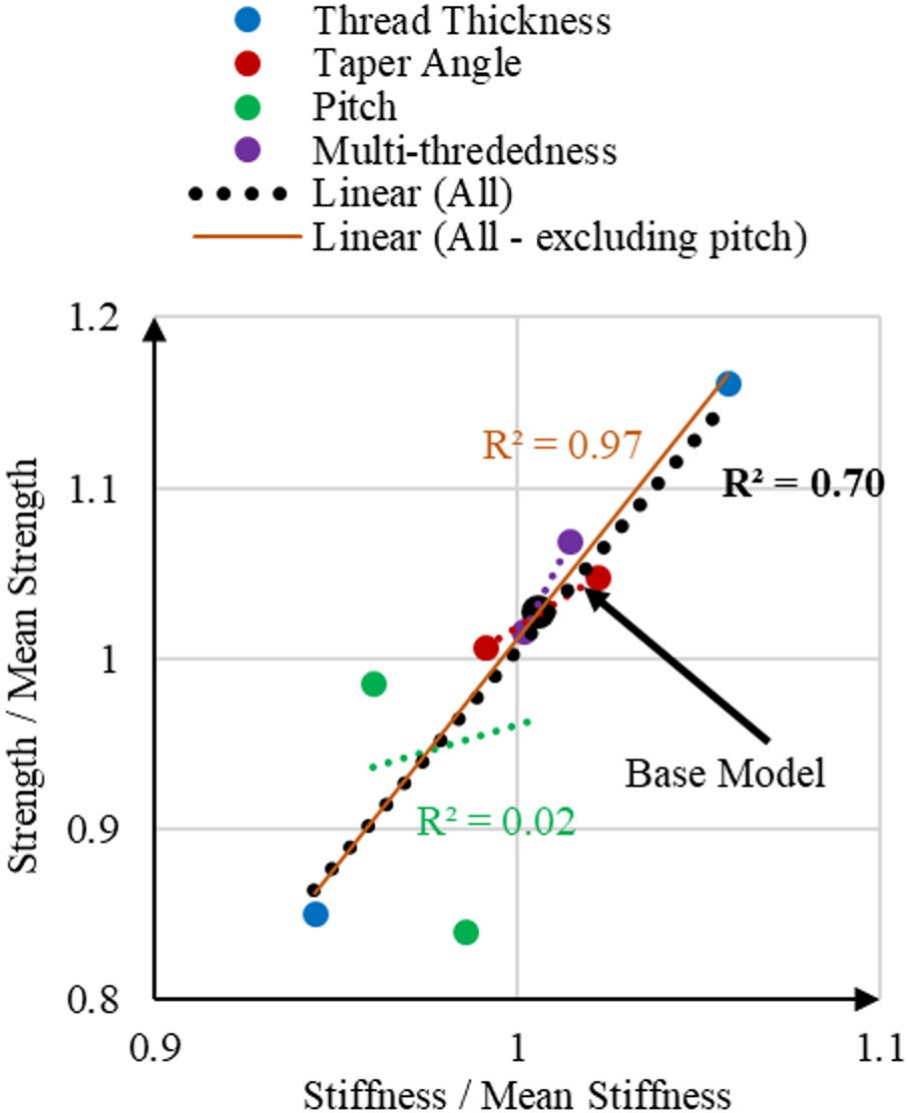
Normalized strength versus normalized stiffness for different values of design parameters.

**Fig. 7:**
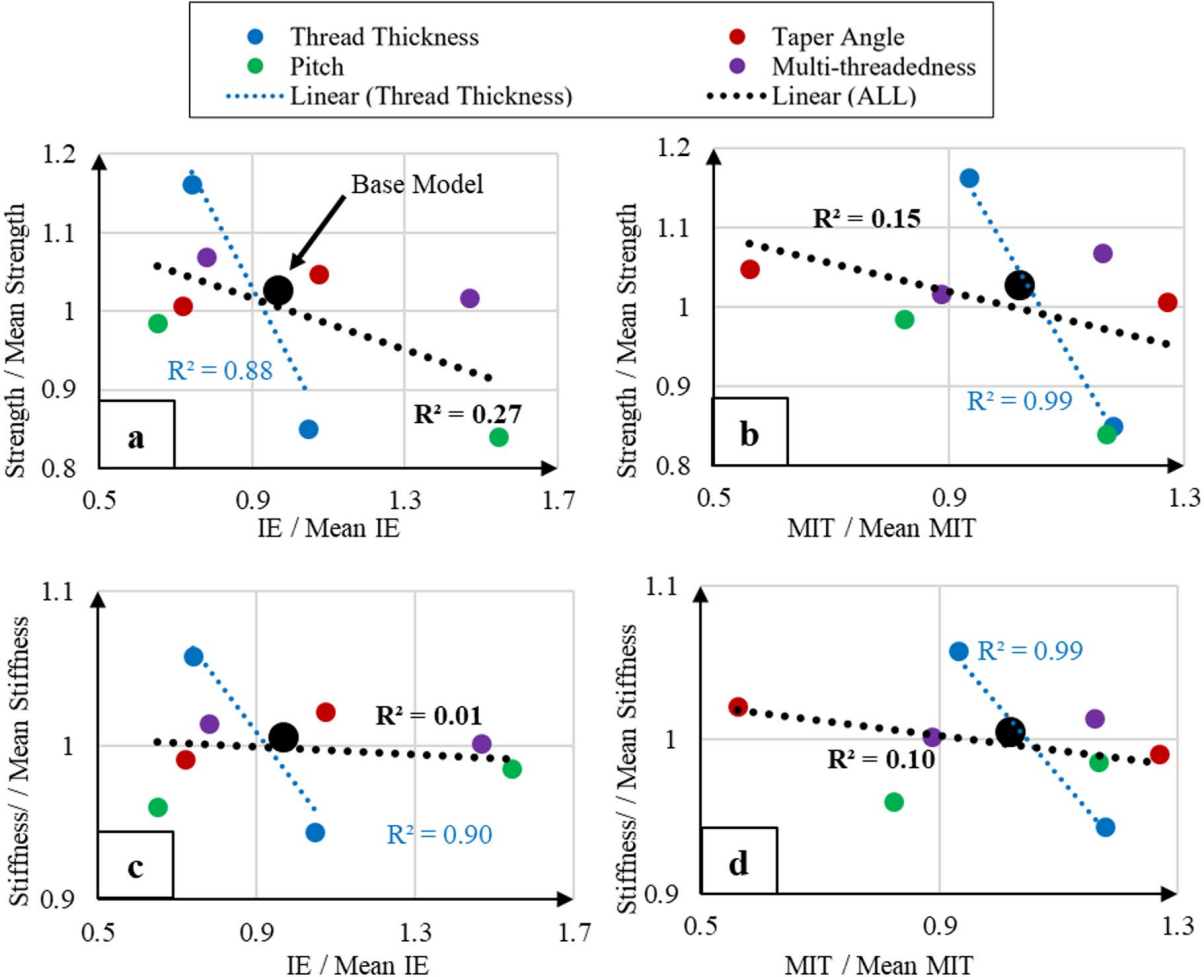
Normalized primary stability indicators obtained from push-in test simulations versus normalized primary stability indicators obtained from insertion process simulations: (**a**) normalized strength versus normalized IE, (**b**) normalized strength versus normalized MIT, (**c**) normalized stiffness versus normalized IE, and (**d**) normalized stiffness versus normalized MIT.

The relationships between the primary stability (PS) indicators investigated in this study are summarized in Figs. 5, 6 and 7. As can be seen in Fig. 5, no meaningful correlation was observed between MIT and IE across all design variations (R² = 0.001). However, within the thread thickness and thread pitch groups, simultaneous increases in MIT and IE were observed, resulting in strong linear relationships (R² = 0.93 and 0.94, respectively).

As shown in Fig. 6, implant–bone construct stiffness and strength exhibited a moderate direct correlation when all design parameters were considered (R² = 0.70). In contrast, variations in thread pitch led to a non‑linear and negligible relationship between stiffness and strength (R² = 0.02). Notably, when the thread-pitch group was excluded, a stronger linear relationship between stiffness and strength was observed (R² = 0.97). Furthermore, Fig. 7 shows that the mechanical stability indicators obtained from the push‑in test, i.e., stiffness and strength, do not exhibit meaningful correlations with the insertion‑process parameters, i.e., MIT and IE. Notably, within the thread thickness group, both stiffness and strength showed inverse relationships with MIT and IE, highlighting a parameter‑specific trade‑off between insertion‑related and post‑insertion stability indicators.

### Design parameters corresponding to the improved model

The selection of design parameters for the improved implant model can be made based on the impact coefficients reported in Table 5, i.e., coefficients of implants primary stability indicators. Since the optimal direction of change for insertion energy (IE), as an indicator of primary stability, remains unclear, i.e., whether a higher or a lower value of it corresponds to a better performance of implantation, this parameter was excluded from the indicators list. Given the strong linear correlation between implant–bone construct stiffness and strength (excluding pitch data points, R² = 0.97, Fig. 6), strength was selected as the representative of the push-in test response, and stiffness was excluded. Moreover, as no meaningful linear relationship was observed between thread pitch variations and bone-implant construct strength (R² = 0.55; Fig. 4c), the thread pitch was kept unchanged at its baseline value. Based on Table 5, it can be observed that the taper angle parameter exerts a positive effect on the MIT with an effect coefficient of approximately + 1.0. Conversely, it has a negative effect on the strength parameter, with an effect coefficient of -0.11. Therefore, selecting the taper angle at its maximum value represents a reasonable trade-off and was chosen as the improved parameter level here. In contrast, the thread thickness parameter exhibited effect coefficients of + 0.35 on MIT and − 1.0 on the strength. Consequently, the thread thickness was set at its minimum value for the optimal implant model. The number of thread starts demonstrated a positive and favorable influence on both MIT and bone-implant construct strength, leading to its selection at the highest level, i.e., the triple-threaded configuration, for the optimized design.

**Fig. 8:**
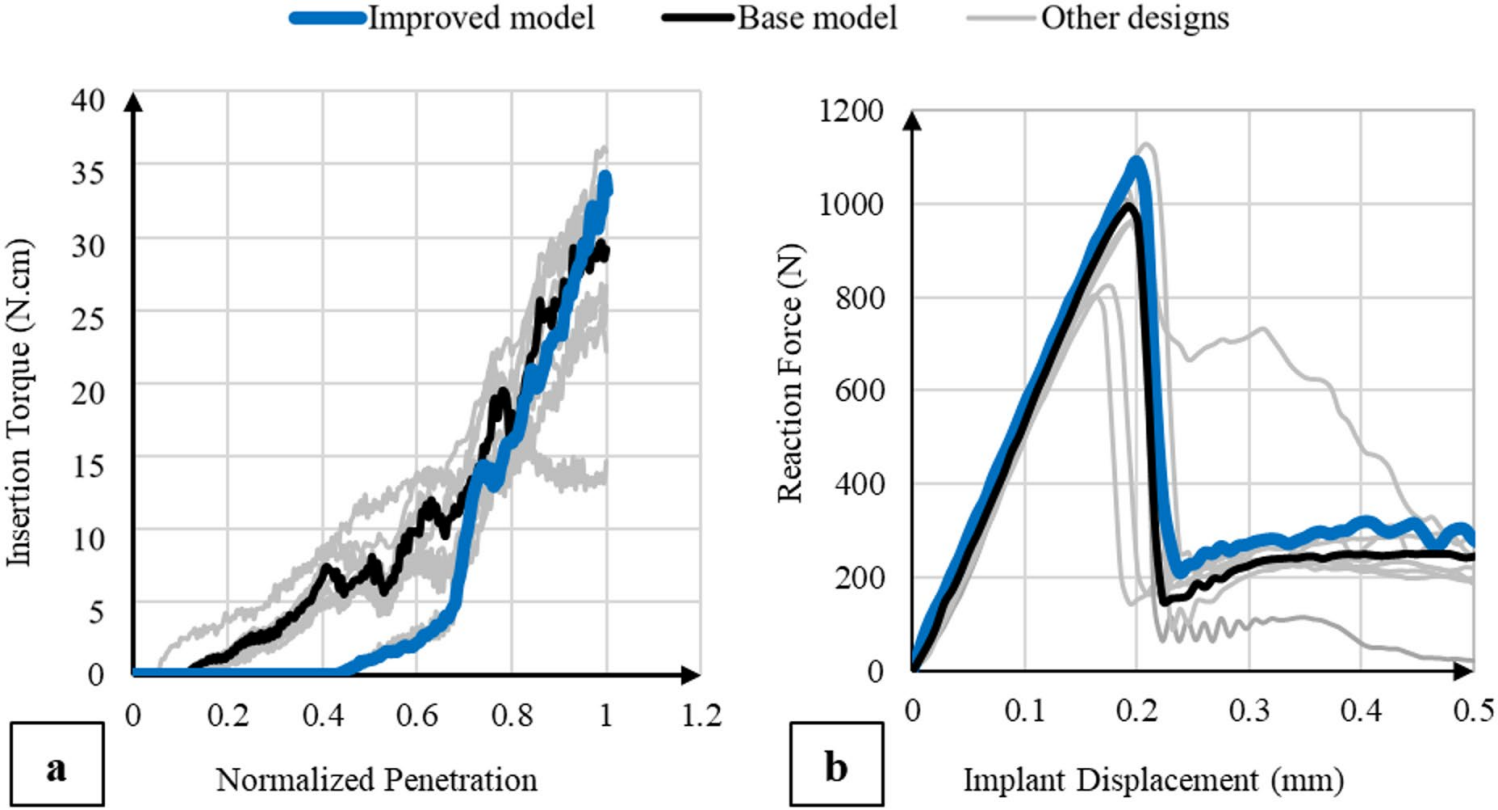
The improved implant design compared with the baseline model and other experimental designs: (**a**) Simulation results of insertion process, and (**b**) Simulation results of push-in test.

**Fig. 9:**
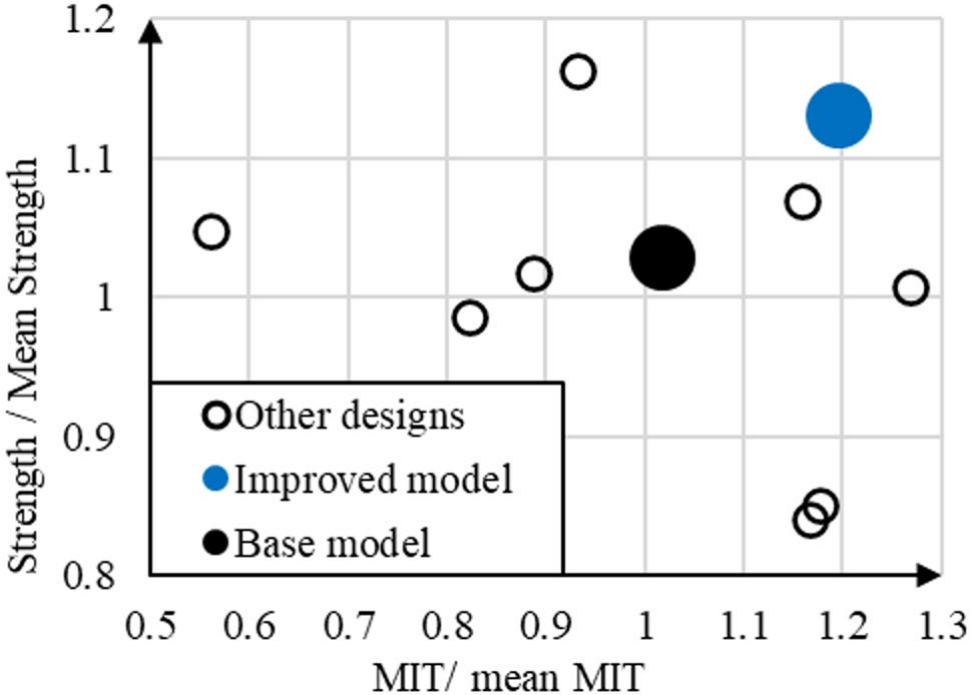
Comparison of the improved implant design versus the baseline design and other designs, based on scatter distribution of normalized strength veruses normalized MIT.

The performance of the chosen improved implant model was evaluated through insertion process and push‑in test simulations, and compared with the baseline design. Figure 8a and b show superiority of the improved model both in regard to MIT and bone-implant construct strength, compared with the base and other models. The combined performance of all designs, illustrated in Fig. 9, shows that the improved model is positioned in the upper‑right region of the MIT–strength space, which indicates a simultaneous improvement in both primary stability indicators. The quantitative values of MIT, IE, bone-implant construct stiffness and strength, along with their percentage changes relative to the baseline model, are summarized in Table 6.

**Table 6:** Evaluation of the improved implant model through comparison with the base implant design and the percentage changes in primary stability indicators.

	PS Indicators			
	Insertion process		Push-in test	
	MIT (N·cm)	IE (J)	Stiffness (kN/mm)	Strength (kN)
Improved Model	34.1	2.61	5.52	1.09
Base Model	29.1	3.89	5.56	0.99
% of Change	17%	-35%	-	10%

## Discussion

In this study, the effects of dental implant design parameters on primary stability (PS) indicators, collected from the in-silico modeling of insertion process and push-in test, were investigated through employing the One Factor At a Time (OFAT) approach. The results showed that different design parameters influenced each PS indicator in a distinct and specific manner. Additionally, it was observed that the PS indicators, as a result of changes made in various design parameters, are interrelated in a variety of ways. These findings highlight the complex relationships between the different design parameters and their combined effects on PS of dental implants.

The results of this work showed that increasing thread thickness and also decreasing thread pitch led to a continuous increase in insertion torque throughout the insertion process (Fig. 4a and b). This behavior can be attributed to the enhanced engagement between the implant threads and the surrounding bone over the entire insertion process, which consequently results in a higher MIT, as reported in some previous studies^37–39^. The sustained increase in thread–bone interaction caused by increasing thread thickness, as well as through reducing thread pitch, also leads to a concurrent increase in IE, as reflected by the larger area under the torque–rotation curve (Fig. 4c; Table 4). In contrast, increasing the number of thread starts and the taper angle resulted in a higher MIT, but a lower IE (Table 4), implying that different design parameters modulate implant–bone mechanics during the insertion process through distinct physical mechanisms.

Consistent with previous experimental studies^24,40^, the present study’s results also showed that conical implants achieve a higher MIT than cylindrical designs (Table 4). Moreover, analysis of the torque–penetration profiles revealed that cylindrical implants exhibited a sharp increase in torque at the onset of insertion process, followed by a near-plateau behavior, whereas conical implants started with a lower torque, but showed a continuous increase toward the final stages of insertion (Fig. 2(a)), in agreement with Wu et al.’ experimental study^24^(also see Supplementary Note: Comparison with some Previous Studies and Supplementary Fig. S3). This behavior can be attributed to the reduced initial thread–bone engagement in conical implants due to their smaller tip diameter, which progressively increases as insertion proceeds. In contrast, in cylindrical implants, engagement with the surrounding bone starts from the beginning of insertion, which results in a more uniform torque evolution and a lower MIT (also see Supplementary Fig. S.4).

Regarding the number of thread starts, the results of this work showed that increasing the number of thread starts reduced the rotations required for a complete insertion, thereby decreases the area under the torque–rotation curve (Fig. 2d), and thus causes a reduction in the IE (Table 4), consistent with experimental study of Yamaguchi et al.^25^(also see Supplementary Note: Comparison with some Previous Studies and Supplementary Fig. S3). However, in contrast to Yamaguchi et al.^25^, and in agreement with Serag Eldeen et al.^41^, the present study showed that double‑ and triple‑threaded implants exhibited higher MITs than single‑threaded designs (Table 4). This behavior can likely be attributed to the greater axial advancement per rotation associated with multi‑threaded designs, which increases resistance during insertion and therefore requires a greater torque. Discrepancies between the present study’s findings and some previous reports (for instance, with^25,41)^ can likely be due to variations in insertion protocols. In previous studies, axial translation was either uncontrolled^25^ or not regulated during manual insertion^41^, whereas in the present simulations, a fixed relationship between rotational- and translational motions was enforced based on the thread lead, ensuring controlled insertion conditions. These results highlight the importance of jointly regulating rotational- and translational parameters when assessing the effect of thread start number on implant insertion.

The results of this work also showed that the taper angle and number of thread starts did not considerably affect the push-in test outcomes (Figs. 3(a) and 3(d), Table 4), which can be attributed to the amount of bone remaining between adjacent threads that cannot be substantially affected by variations in taper angle or by number of thread starts. In contrast, thread thickness and pitch had a notable influence on the push-in response (Figs. 3(b) and 3(c), Table 4). As reported in previous studies^29,42^, the bone trapped between the threads determines the resistance of implant displacement during the push-in test. Consistent with this mechanism, the present study showed that increasing thread thickness reduces the volume of bone trapped between threads, which leads to a lower stiffness, as well as strength of the implant–bone construct (Table 4).

It was observed in this study that thread pitch exhibited a non‑monotonic effect on the push‑in response, such that both increasing and decreasing the pitch resulted in a reduced stiffness and strength of the implant–bone construct (Fig. 3c; Table 4). The reduction in stiffness and strength observed here with decreasing pitch is expected, as it results from the diminished volume of bone remaining between adjacent threads. However, by increasing the pitch, it was expected to see an increase in both stiffness and strength, as it produces a larger inter‑thread bone volume. This counterintuitive behavior can likely be attributed to the simultaneous reduction in the number of threads and, consequently, in the effective implant–bone contact area. This reduction in the contact area may promote localized stress concentrations, thereby eliminating the mechanical benefits of the increased remaining bone volume. These findings indicate that implant stability during push‑in loading is governed jointly by the volume of remaining bone and the effective contact area, and cannot be explained by inter‑thread bone volume alone.

From a clinical viewpoint, a higher MIT is commonly considered desirable for immediate loading implantation^5,14^, although excessive torque may induce bone damage^21,23^ or marginal bone loss^43^. On the other hand, Ovesy et al.^20^ reported that the increase in MIT resulting from undersizing the pilot hole did not significantly affect the stiffness of the implant–bone construct. Similarly, the present study’s results showed that a higher MIT does not necessarily translate into a greater stiffness or strength, and may instead weaken the implant–bone construct, in some cases (Table 4). For example, increasing thread thickness or decreasing thread pitch increased MIT, but reduced construct stiffness and strength (Table 4). On the other hand, increasing taper angle or the number of thread starts caused an increase in MIT, with negligible effects on push-in outcomes (Table 4). These observations indicate that the biomechanical implications of increased MIT are strongly dependent on the underlying cause of this increase. Therefore, it is essential to identify the factors responsible for elevated MIT and to evaluate their effects on other primary stability indicators. Consequently, an increase in MIT alone cannot be considered a robust or sufficient criterion for assessing implant performance.

Regarding the relationship between MIT and implantation success rate, systematic reviews and meta-analyses have shown that higher MIT values are not consistently associated with improved clinical outcomes^44,45^, and lower values are not necessarily linked to implantation failure^46^. This is notable given the widespread use of MIT as an important clinical indicator of implant performance. Although the present study is in-silico, mechanical in nature, the findings suggest that the factors responsible for increasing MIT may influence other aspects of mechanical behavior differently. For instance, changes in thread thickness and taper angle both increased MIT in this work, yet their effects on push-in mechanical response were in the same direction. These observations indicate that similar MIT values may arise from different mechanical conditions at the bone–implant interface, which could influence the biology differently in both the short- and long-term periods, following insertion of the implant. This perspective may partly explain why clinical studies have not consistently reported a clear relationship between MIT magnitude and implant success^44–46^.

To date, some researchers have proposed insertion energy (IE) as an indicator of PS^8,40,47^. Moreover, while several studies have reported strong correlations between MIT and IE^8,40^, others have shown that these parameters may vary independently, depending on implant design^9^ and surgical conditions^29^. The results of the present study showed that there is no overall linear relationship between MIT and IE across all experimental groups (R² = 0.01; Fig. 5). However, within specific subgroups defined by thread thickness and thread pitch, a strong linear relationship between IE and MIT was observed (R² = 0.99 and 0.82, respectively; Fig. 5). These findings indicate that MIT and IE capture different aspects of the insertion process. Specifically, MIT reflects a localized peak resistance occurring at a particular stage of the non‑reversible process of insertion, whereas IE represents the cumulative resistance integrated over the entire insertion path.

The absence of a consistent overall relationship among the PS indicators examined in this study highlights the multifactorial and sometimes conflicting nature of implant–bone mechanical interactions. For example, previous studies have suggested increasing thread thickness^39^, or decreasing thread pitch^38^ to enhance PS in terms of MIT, a trend also confirmed in the current study (Figs. 2(b) and 2(c), Table 4). However, results of this work further demonstrated that increasing thread thickness and decreasing thread pitch adversely affect the stiffness and strength of the implant–bone construct (Figs. 3(b) and 3(c), Table 4), highlighting the inherent limitations of assessing PS based on a single indicator. Despite the absence of general linear relationships among the PS indicators (Figs. 5, 6 and 7), the improved model presented in this study demonstrates that a clear understanding of the interactions between stability indicators and their governing design parameters enables strategic adjustment of implant geometry, leading to the simultaneous improvement of multiple PS indicators. This integrated perspective suggests a paradigm shift toward treating insertion and post‑insertion responses as interconnected components of a unified biomechanical system within a smart and adaptive host environment^48^.

This study suffers from some limitations that should be acknowledged here. First, bone was modeled as a homogeneous, isotropic, and linear elastic–plastic material with ductile damage, whereas real bone exhibits heterogeneous and anisotropic behavior. In particular, even though trabecular bone is an anisotropic material with a non-linear material characteristic, for computational efficiency and consistency with many previous numerical implant studies^27,29,32^, its elastic phase was represented here using a single constant elastic modulus. Thus, the adopted material model may not fully capture the directional mechanical behavior of trabecular bone. In addition, the Young’s modulus assigned to trabecular bone represents an idealized value that may be higher than that of some clinical conditions, which should be considered when interpreting the quantitative results. Also, bone fracture was simulated using an element deletion technique, while in reality, fractured bone fragments may remain between the implant and surrounding tissue and influence the insertion process. Furthermore, as it is the case with other finite element analyses, the present simulations cannot fully replicate the biological and mechanical complexities of the in vivo oral environment, such as patient-specific bone morphology, biological remodeling, and surgical variability. Therefore, the findings of this study should be interpreted with sufficient care. Second, the OFAT experimental design with three levels per parameter was employed in this study, which limits the assessment of nonlinear effects and interactions among design variables. Nevertheless, the conceptual insights gained from this work, most notably the multidimensional nature of PS and the proposal of improved implant design based on the independent evaluation of multiple PS indicators, offer a new perspective in this field of research. Future studies incorporating multifactorial design strategies along with in vitro and in vivo experimental validations are necessary to further substantiate the findings reported here and help researchers develop more efficient dental implant designs.

## Conclusions

This study has demonstrated that each dental implant design parameter influences the insertion process, as well as push-in test in distinct and intricate ways, with a different impact on primary stability (PS) indicators, such as maximum insertion torque (MIT), insertion energy (IE), and bone-implant construct stiffness and strength. It was also observed here that a higher MIT does not necessarily correlate with a better mechanical performance, such as an enhanced bone-implant construct stiffness and strength. For instance, increasing MIT through considering a greater thread thickness, or a smaller thread pitch has a negative impact on bone-implant stiffness and strength, and reduces both. This finding implies the complexity of the relationship between MIT and mechanical characteristics, highlighting that a higher MIT does not always result in a greater success rate of implantation. Moreover, the results from the improved implant model found here showed that by analyzing the effects of each design parameter on different PS indicators individually, it is possible to optimize the design in such a way that most PS indicators can be simultaneously enhanced. The improved implant model proposed here successfully enhanced both the MIT and the strength of the bone-implant construct simultaneously, so it can provide useful insights for future implant design strategies. The approach proposed in this study provides a novel perspective in the optimization of dental implant designs, where both the insertion process and mechanical tests, such as push-in test, can be integrated into a unified framework.

## Supplementary Information

Below is the link to the electronic supplementary material.

Supplementary Material 1

## Data Availability

The datasets generated during and/or analysed during the current study are available from the corresponding author on reasonable request.
