# Supplementary material for: Improving dental implant design through a combined analysis of the insertion process and push-in test
Source: Sci Rep. 2026 May 18;16:22476. doi: 10.1038/s41598-026-53352-3 (PMC13376155; doi:10.1038/s41598-026-53352-3)
Supplement: Supplementary file 1 — Supplementary Material 1 [file 41598_2026_53352_MOESM1_ESM.pdf]

supplementary Information

For the article*: "Improving Dental Implant Design Through a Combined Analysis of the Insertion Process and Push-In Test"*

Supplementary Note: Mesh Independence Analysis

Supplementary Fig. S.1 presents the torque-penetration taken from insertion process simulations for the base implant model using mesh sizes of 200, 100, 50, and 25 µm. As can be observed the finite element model developed here exhibits a relatively high dependency on the mesh size. This dependency is likely due to the nonlinear nature of bone damage, the implant–bone surface engagement, and the element deletion technique employed in the current work. Further insights can be drawn from Supplementary Fig. S.2, which illustrates the implant–bone construct after the insertion process and prior to the push-in test for different mesh sizes. As the mesh size decreases, the bone tissue surrounding the implant can more accurately fill the spaces between the implant threads. For instance, in a mesh model with the size of 200 µm, only three bone elements remain between the threads, and if design parameters, such as thread thickness changes by 0.1 mm, then the model lacks sufficient precision to capture performance differences. Ultimately, based on the acceptable coverage of implant–bone engagement and a deviation of less than 5 % in MIT and IE, compared to the model with a mesh size of 25 µm, a mesh size of 50 µm was selected for all simulations in this study.

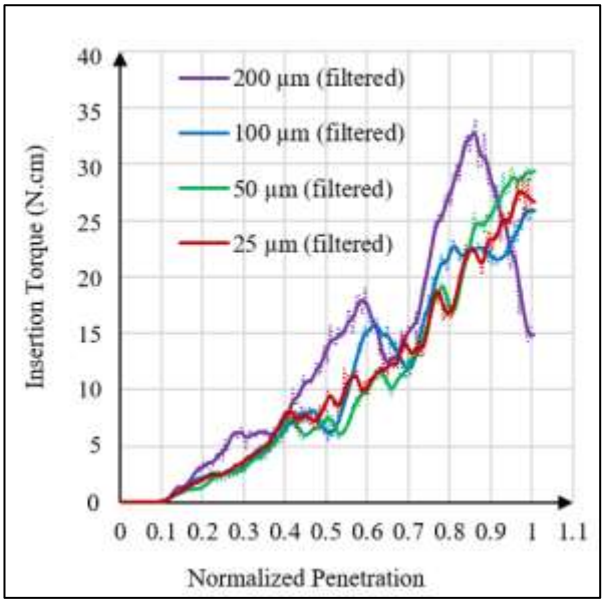

Supplementary Figure S.1: Changes in the insertion torque versus penetration for the base sample for different mesh sizes

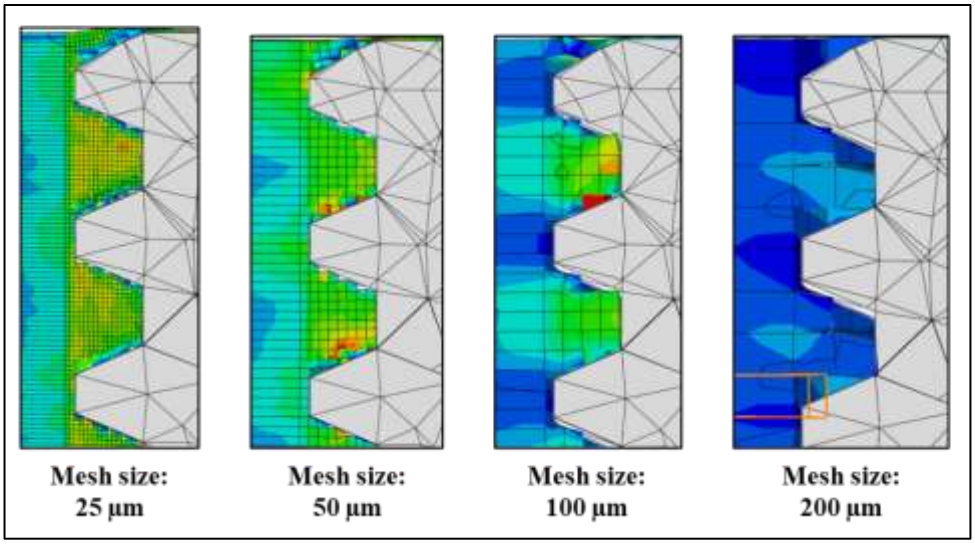

Supplementary Figure S.2: Differences in mesh arrangements with different mesh sizes after the insertion process

Supplementary Note: Implant Lead and Rotation During Insertion

Since the implant designs investigated in this study differed in thread lead, the total rotational displacement required to achieve a full insertion depth of 10 mm was not identical across all models. Thread lead is defined as the axial advancement of the implant during one complete rotation and is determined by the product of the thread pitch and the number of thread starts. Consequently, implants with different thread configurations require different total rotations to reach the same insertion depth. The thread pitch, the corresponding calculated lead, and the total rotation required for complete insertion of each implant design are summarized in Supplementary Table S.1.

Supplementary Table S.1: Thread pitch, lead, and required rotation for implant insertion for all implant designs investigated in this study.

	Pitch (mm)	Lead (mm)	Rotation for insertion (radian)
**Base Model**	0.8	1.6	39.27
**Lower TA**	0.8	1.6	39.27
**Higher TA**	0.8	1.6	39.27
**Lower TT**	0.8	1.6	39.27
**Higher TT**	0.8	1.6	39.27
**Lower TP**	0.55	1.1	57.12
**Higher TP**	1.05	2.1	29.92
**Single-Threaded**	0.8	0.8	78.54
**Triple-Threaded**	0.8	2.4	26.18

Supplementary Note: Mesh Characteristics

Supplementary Table S.2 summarizes the mesh characteristics of all finite element models employed in this study, including the base model, the higher and lower taper-angle (TA) configurations, the higher and lower thread-thickness (TT) models, the higher and lower thread-pitch (TP) models, the single- and double-threaded designs, and the models with different numbers of thread starts (TS). For each geometrical model, the table reports the element type, the total number of elements, and the total number of nodes used in the discretized geometry.

Supplementary Table S.2: Mesh characteristics of all finite element models used in this study for the implants and bone

	Mesh Characteristics		
	**Element Type**	**# of Nodes**	**# of Elements**
**Base Model**	10-node quadratic tetrahedral	35229	23037
**Lower TA**	10-node quadratic tetrahedral	39028	25666
**Higher TA**	10-node quadratic tetrahedral	28662	18454
**Lower TT**	10-node quadratic tetrahedral	36725	24124
**Higher TT**	10-node quadratic tetrahedral	36581	24202
**Lower TP**	10-node quadratic tetrahedral	40457	26402
**Higher TP**	10-node quadratic tetrahedral	31274	20467
**Single-Threaded**	10-node quadratic tetrahedral	33620	22069
**Triple-Threaded**	10-node quadratic tetrahedral	37528	24548
**All Bone Models**	8-node linear hexahedral	250848	228800

Supplementary Note: Comparison with some Previous Studies

In Supplementary Figures S.3a and S.3b, the results related to single-threaded and double-threaded implants are presented for the present study and for the study by Yamaguchi et al. [1], respectively. Consistent with the findings of Yamaguchi et al. [1], the present study also demonstrates that the number of implant rotations during insertion for double-thread implants is approximately half that of single-thread implants. This reduction in the number of rotations leads to a lower insertion energy for multi-thread implants compared with single-thread implants, as a result of the decreased area under the rotation–insertion torque curve.

Supplementary Fig. s.3d shows the experimental results of Wu et al. [2], where the difference in the insertion process between cylindrical and conical implants was studied. The results of the present study in regard to variation of torque versus penetration depth for cylindrical (0° taper angle) and conical implants (3° and 6° taper angles) are in agreement with those of Wu et al. [2] (Supplementary Figs. S.3c and S.3d). As can be seen, conical implants require less torque at the initial stages of insertion, compared to cylindrical implants. However, as the insertion process

continued, the torque increased, and ultimately, the maximum torque required for conical implants is greater than that of cylindrical implants (Supplementary Figs. S.3c and S.3d).

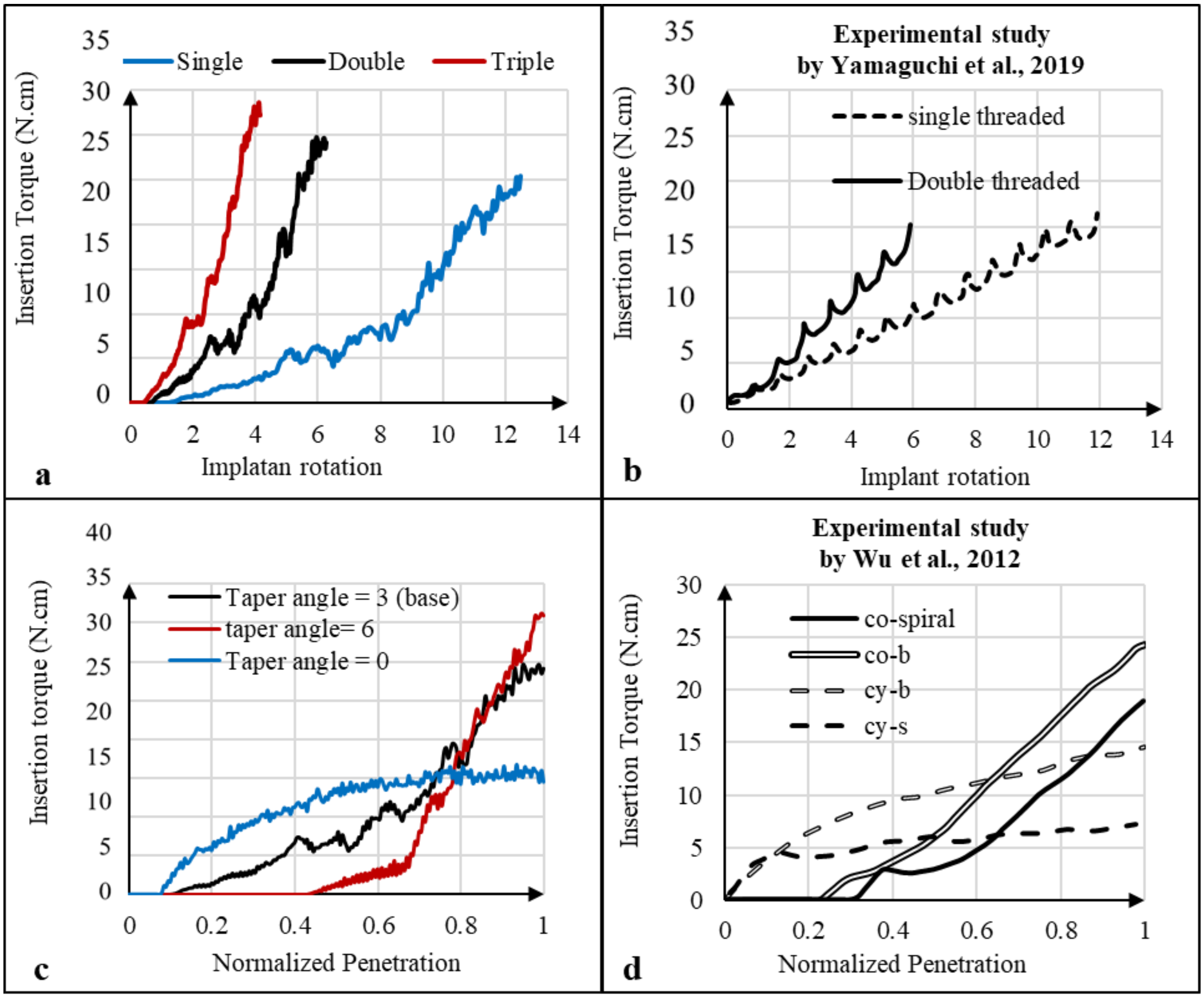

Supplementary Figure S.3: Comparison of some of the insertion process results from the current in-silico study with some previous experimental investigations: (a) Results from the multi-threaded group of the current study, (b) Results from Yamaguchi et al. (2019), (c) Results from the taper angle group of the current study, and (d) Results from Wu et al. (2012)

Supplementary Note: Stress contours during insertion for the taper angle group

Supplementary Figure S.4 shows the evolution of von Mises stress distribution in bone during insertion for implants with taper angles of 0°, 3°, and 6°. As the taper angle increases, implant–bone engagement is reduced during the early phase of insertion, whereas thread engagement occurs more in the later phase. This can be observed by comparing the 0° and 6° taper angles' designs at the 20% of insertion frame. In the cylindrical implant of taper angle, thread engagement begins early, producing measurable bone stress, and in later stages of insertion (i.e., 60% and 100%), the threads of the cylindrical implant advance mainly within the path which was already created by

the preceding threads, resulting in a limited additional bone engagement. In contrast, for the 6° taper design, multiple threads simultaneously contact the walls of the pilot hole and penetrate the surrounding bone, which lead to a more simultaneous implant–bone engagement during the insertion process.

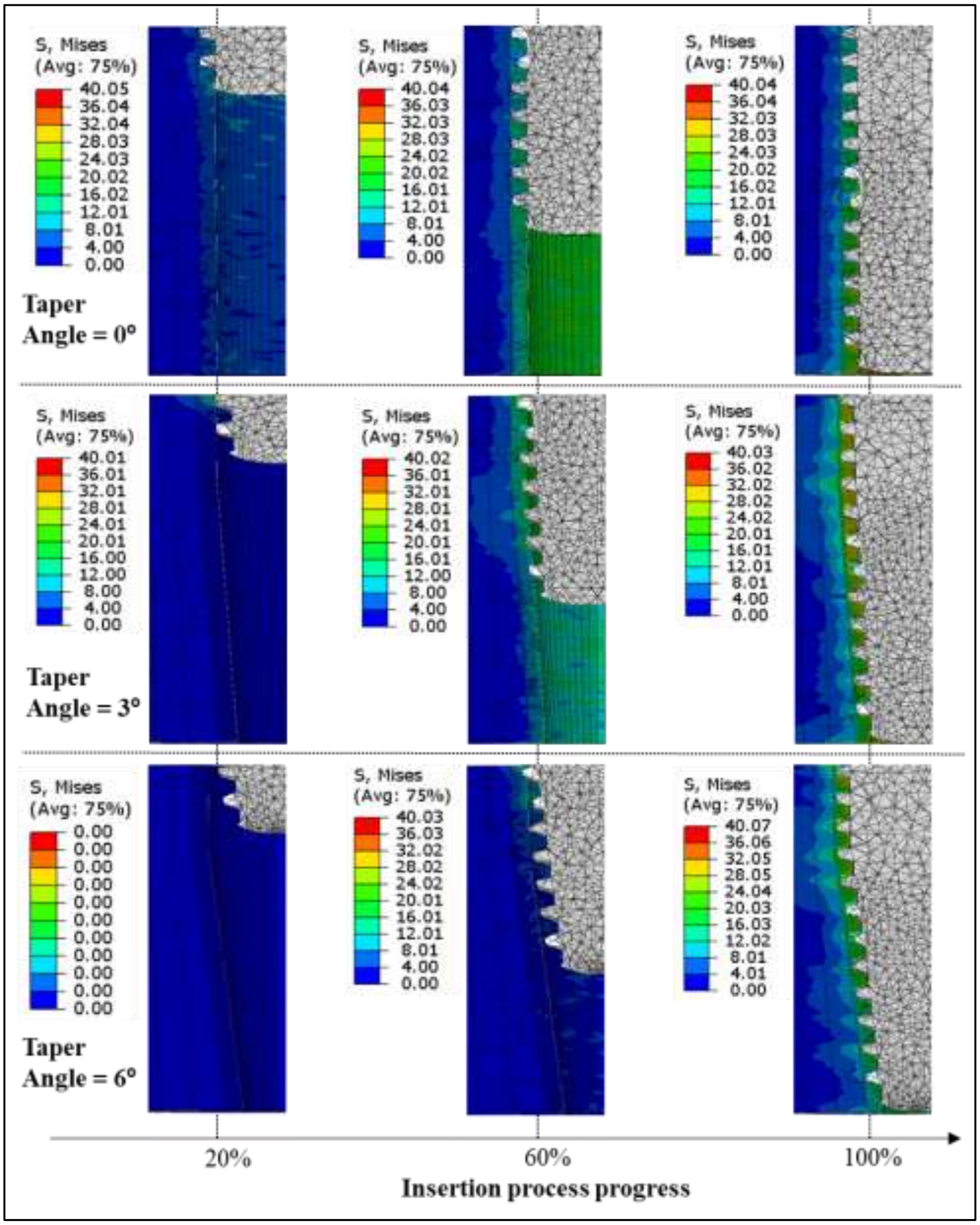

Supplementary Figure S.4: von Mises stress distribution in the surrounding bone during implant insertion for implants with taper angles of 0°, 3°, and 6°, shown at 20%, 40%, and 100% of the insertion process.

References

[1] Y. Yamaguchi, M. Shiota, M. Fujii, M. Shimogishi, M. Munakata, Effects of implant thread design on primary stability—a comparison between single- and double-threaded implants in an artificial bone model, Int J Implant Dent 6 (2020) 42. https://doi.org/10.1186/s40729-020-00239-1.

[2] S.-W. Wu, C.-C. Lee, P.-Y. Fu, S.-C. Lin, The effects of flute shape and thread profile on the insertion torque and primary stability of dental implants, Med Eng Phys 34 (2012) 797–805. https://doi.org/10.1016/j.medengphy.2011.09.021.
